# *Bartonella*-associated glomerulonephritis without endocarditis in a renal transplant recipient

**DOI:** 10.1128/asmcr.00092-25

**Published:** 2025-10-07

**Authors:** Grace A. Schaack, Rene Bulnes, Deborah Jebakumar, Allen R. Hendricks, Andrew E. Clark

**Affiliations:** 1 Department of Pathology, University of Texas Southwestern Medical Center 465773, Dallas, Texas, USA; Rush University Medical Center, Chicago, Illinois, USA

**Keywords:** *Bartonella*, glomerulonephritis, bartonellosis, cat scratch disease, culture-negative, kidney, case report

## Abstract

**Background:**

*Bartonella henselae* is a fastidious gram-negative rod that can cause systemic disease in immunocompromised patients. Infection may be complicated by glomerulonephritis, often in the setting of culture-negative infective endocarditis. The histologic pattern of *Bartonella*-associated glomerulonephritis is variable and often difficult to distinguish from non-infectious causes of glomerulonephritis by histopathology. Prompt, accurate diagnosis is further complicated by the poor sensitivity of routine culture methods for the detection of *B. henselae*, and serology or adjunctive molecular testing is typically required for microbiologic diagnosis.

**Case Summary:**

A middle-aged male renal transplant recipient was admitted to our hospital with fever, nausea, vomiting, diarrhea, and laboratory evidence of acute kidney injury. A thorough infectious disease work-up revealed markedly elevated *B. henselae* immunoglobulin G and immunoglobulin M titers and significant exposures to cats and fleas. A biopsy of the renal allograft revealed crescentic glomerulonephritis with immune complex deposition. No definitive evidence of infective endocarditis was found. The patient was started on renal replacement therapy and treated for disseminated bartonellosis with doxycycline and rifabutin.

**Conclusion:**

The diagnosis of *Bartonella*-associated glomerulonephritis in immunocompromised patients can be challenging from both the clinical and histopathologic perspectives. A high degree of clinical suspicion in patients with relevant risk factors is important for prompt diagnosis and treatment. Regardless of the presence or absence of typical signs and symptoms of cat scratch disease and/or infective endocarditis, *Bartonella* spp. should be included in the differential diagnosis of glomerulonephritis in solid-organ transplant recipients and other immunocompromised patients with cats or other relevant exposures.

## INTRODUCTION

*Bartonella henselae* is a species of fastidious, gram-negative, pleomorphic, rod-shaped bacteria that is well-known as the causative agent of cat scratch disease (1, 2). *Bartonella* species are facultatively intracellular pathogens that primarily infect host erythrocytes and endothelial cells. They can infect numerous host species and are transmitted by a variety of hematophagous arthropod vectors. In the United States, the classic reservoir and vector for *B. henselae* are the cat and the cat flea (*Ctenocephalides felis*), respectively. In feline hosts, *B. henselae* replicates intraerythrocytically, facilitating immune evasion and bacteremia. Kittens are more likely to transmit *B. henselae* than adult cats, and it is estimated that approximately half of all domestic cats are or have been infected (3). Humans can become infected with *B. henselae* upon contact with cats and/or cat fleas and are a dead-end host (1).

Infection with *B. henselae* in humans typically manifests as cat scratch disease characterized by erythematous papular skin lesions at the site of inoculation, regional lymphadenopathy, fever, and fatigue (1). In immunocompromised hosts, infection is more likely to cause disseminated bartonellosis, and these patients may lack the characteristic skin lesions and lymphadenopathy seen in typical cat scratch disease (4, 5). In a review of 29 cases of *B. henselae* infections in solid organ transplant recipients, Psarros et al. found that the majority (72%) of these patients developed disseminated disease, lymphadenopathy was present in 41% of the cases, and skin lesions were present in only 24% of the cases (4).

Glomerulonephritis is an important complication of disseminated bartonellosis that can be challenging to accurately attribute to *Bartonella* infection, particularly in immunocompromised patients lacking a classical presentation of cat scratch disease. A variety of histologic patterns of glomerulonephritis may be seen on renal biopsy in the setting of bartonellosis (6–15). This variability in both clinical and histopathologic presentation makes *Bartonella* infection difficult to distinguish from other infectious and non-infectious causes of glomerulonephritis. *B. henselae* is also an important etiologic agent of culture-negative infective endocarditis, and *Bartonella*-associated glomerulonephritis often occurs within this setting. Rarely, however, glomerulonephritis due to *Bartonella* infection has been reported in the absence of definitive evidence of endocarditis (16–18). Here, we present a case of disseminated bartonellosis with glomerulonephritis in a renal transplant patient without definite endocarditis and without classic features of cat scratch disease, highlighting the importance of considering *B. henselae* infection in immunosuppressed individuals with relevant environmental exposures.

## CASE PRESENTATION

A middle-aged male kidney transplant recipient presented to the emergency department for further evaluation after having been found to have elevated creatinine levels during a clinic visit. He reported an approximately 3-week history of malaise and watery, non-bloody diarrhea with worsening nausea and vomiting. He also reported low blood pressure readings at home, subjective fevers, and unquantified weight loss.

His past medical history was pertinent for type 1 diabetes mellitus complicated by end-stage renal disease, for which he received a deceased-donor kidney transplant. His transplant was performed 18 weeks prior to presentation and was complicated by delayed graft function. He underwent induction therapy with rabbit anti-thymocyte globulin, and his maintenance immunosuppression included mycophenolic acid, tacrolimus, and prednisone. His antimicrobial prophylaxis consisted of trimethoprim-sulfamethoxazole and valacyclovir. He resided in a house in Texas with multiple cats. He also recalled a recent flea infestation of his home, presumably attributable to his recent adoption of two rescued kittens and his numerous other cats with indoor and outdoor access.

Upon presentation, he had a fever of 38.6°C, dry oral mucosa, no abdominal distension or tenderness, and multiple lacerations on his hands and feet inflicted by his cats. No other skin findings were present. Lymphadenopathy was absent. Initial laboratory studies were notable for thrombocytopenia (86 × 10^3^/µL), hyponatremia (121 mM), hypokalemia (3.3 mM), elevated serum creatinine (5.84 mg/dL whereas at baseline 2.1 mg/dL) and hypocomplementemia (C4 and C3 levels of 13 mg/dL). He was admitted for management of acute kidney injury and was empirically started on ceftriaxone and vancomycin. Doxycycline was added to the empiric regimen for rickettsial coverage in the setting of immunosuppression and geographic risk factors.

Initial microbiologic studies, including blood cultures, stool cultures, stool examination for ova and parasites, and stool PCR via FilmArray GI Panel (BioFire Diagnostics, Inc.) were negative. Additional tests for Q fever, murine typhus, coccidioidomycosis, histoplasmosis, bartonellosis, and toxoplasmosis were ordered. On the ninth day of admission, serologic testing revealed an anti-*B*. *henselae* IgG titer of 1:8192 (normal < 128), anti-*B*. *henselae* IgM ≥ 1:20 (normal < 20), and anti-*B*. *quintana* IgG titer of 1:512 (normal < 128). Targeted PCR for *Bartonella* in a whole-blood specimen was negative. Rifabutin was initiated along with the continuation of doxycycline for the treatment of bartonellosis. A metagenomic sequencing assay using cell-free DNA from plasma (Karius Inc.) detected DNA from *B. henselae,* but this result returned after patient discharge.

Cardiac imaging by both transthoracic and transesophageal echocardiogram did not reveal any valvular vegetations. Computed tomography (CT) of the abdomen and pelvis identified mild splenomegaly (13.5 cm) and two nonspecific peripheral splenic lesions (2 and 2.7 cm in diameter) that were not investigated further. Additional studies, including chest CT, esophagogastroduodenoscopy, and colonoscopy, did not reveal evidence of direct involvement by *Bartonella* infection.

**Fig 1:**
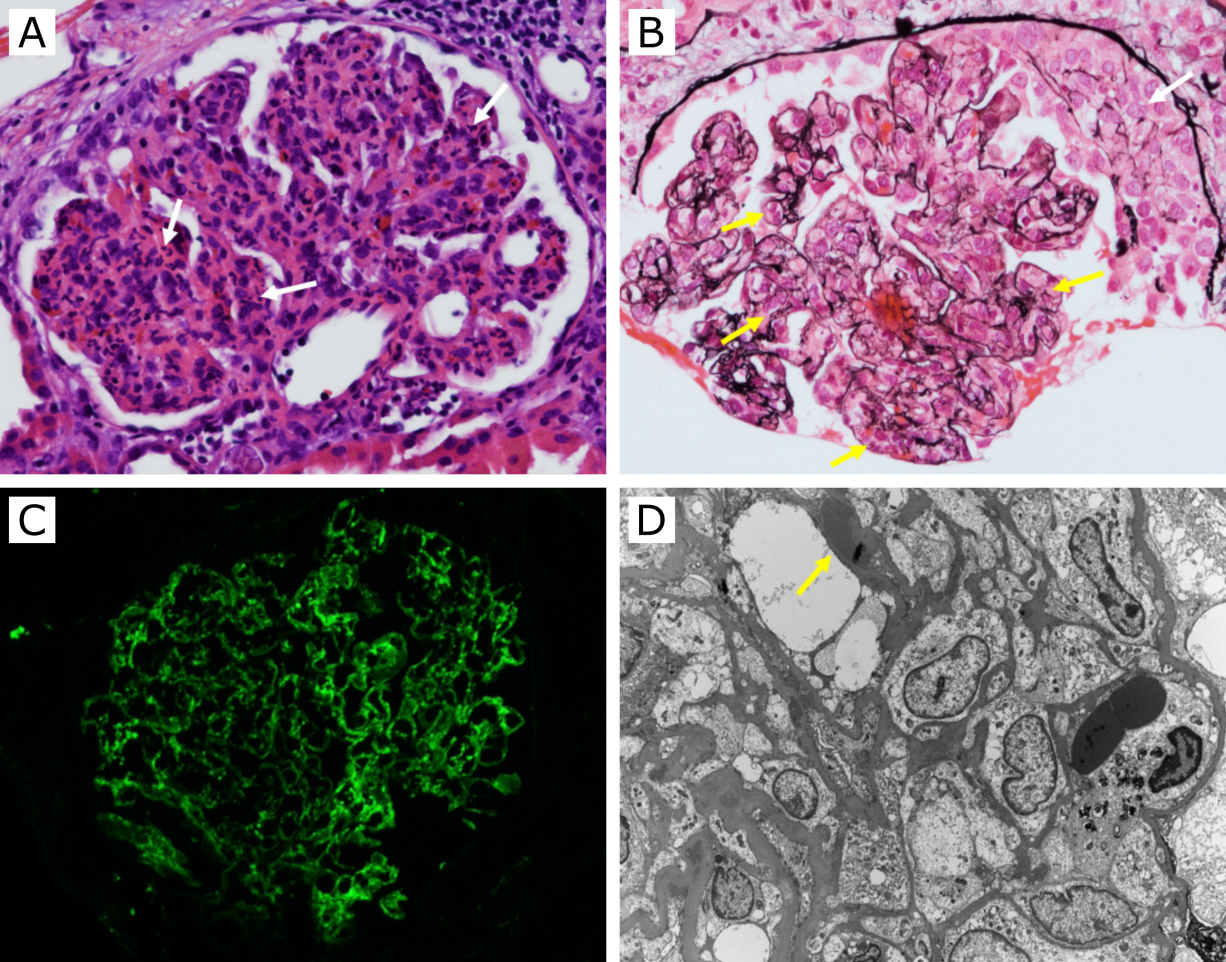
Kidney biopsy. (**A**) H&E stain shows numerous neutrophils within a glomerulus (white arrow, ×200). (**B**) Jones methenamine silver stain shows global endocapillary hypercellularity (yellow arrows) and a cellular crescent (white arrow, ×200). (**C**) Immunofluorescence for C3 shows diffuse, segmental, granular glomerular staining (×200). (**D**) Electron microscopy shows severe foot process effacement and multiple subepithelial electron-dense deposits, some of which are hump-shaped (yellow arrow, ×2,550).

On the 14th day of admission, a biopsy of the renal allograft was performed and showed evidence of glomerulonephritis. Fifteen open glomeruli showed diffuse, global endocapillary hypercellularity with numerous neutrophils (Fig. 1A). Two glomeruli had cellular crescents (Fig. 1B). Moderate tubulitis and severe acute tubular injury were present with multifocal interstitial edema and moderate inflammatory cell infiltration by mononuclear leukocytes and small numbers of eosinophils. Immunofluorescence microscopy showed diffuse, segmental, granular glomerular capillary wall and mesangial positive staining by immunoglobulin A (IgA) (4 + on a 0–4 scale), immunoglobulin M (IgM) (4+), C3 (4+, Fig. 1C), C4d (4+), kappa (3+), and lambda (3+). Ultrastructural examination of two glomeruli showed severe (>80%) foot process effacement. Multiple subepithelial, subendothelial, and mesangial immune-complex-type, electron-dense deposits were seen. Some of the subepithelial deposits were “hump-shaped” (Fig. 1D). No tubuloreticular inclusions were identified. These findings were consistent with an IgA-codominant, infection-related glomerulonephritis.

The patient’s gastrointestinal symptoms and fever resolved after 4 days; however, his kidney function did not recover in the short term, and he required intermittent renal replacement therapy. He ultimately completed a 2-week course of rifabutin and 3 months of doxycycline along with a prednisone taper. By the time antibiotic therapy was completed, his renal function stabilized and he no longer required dialysis.

## DISCUSSION

This case illustrates several potential challenges in the diagnosis of *Bartonella*-associated glomerulonephritis. The patient’s immunocompromised status and exposures to cats and fleas were key elements of the history that led to early consideration of *Bartonella* infection despite the absence of a typical presentation of cat scratch disease. Because *Bartonella* species are not typically detected in routine culture, a high index of suspicion is required to ensure appropriate diagnostic test selection and correct interpretation of microbiologic and histopathologic data.

*B. henselae* culture is challenging, requiring extended incubation times of up to 21 days at 35–37°C in 5% CO_2_ (2). Due to the low sensitivity of culture-based methods, laboratory diagnosis relies heavily on serology (19–21). Serologic testing is currently the most reliable, sensitive, minimally invasive testing method for *B. henselae*, but it is important to be mindful of its limitations when interpreting results (20, 21). For example, serologic assays for *Bartonella* exhibit cross-reactivity with *Chlamydia*/*Chlamydophila* and with *Coxiella* (20). There is also serological cross-reactivity between *B. henselae* and *B. quintana*, but markedly higher titers against one species versus the other are indicative of the more likely causative agent (20). It is also notable that mildly elevated titers may be present at baseline in the general healthy population, so it is important to look for evidence of true positivity (IgG titer of at least 1:256 and/or a fourfold rise in IgG titer) through comparison of acute and convalescent samples (21). In the case presented here, the dramatically elevated anti-*B*. *henselae* IgG titer and mildly elevated anti-*B*. *quintana* IgG titer are suggestive of a true *B. henselae* infection resulting in production of IgG with mild cross-reactivity against *B. quintana*.

In addition to serology, complementary testing via histology and nucleic acid amplification and sequencing methodologies can be helpful for diagnosis (20, 21). If biopsy specimens are available, histologic examination using immunohistochemistry or nonspecific methods such as Warthin-Starry silver impregnation can help to directly visualize organisms in tissue. Targeted nucleic acid amplification testing and broader-range sequencing approaches (i.e., 16S rRNA sequencing and shotgun metagenomic approaches) may be used to identify *B. henselae* in patient specimens but show better sensitivity for solid tissue specimens than for blood (21). Therefore, while a positive *Bartonella* PCR result is supportive of a diagnosis of bartonellosis, a negative PCR result, as was seen in our case, is by no means contrary to the diagnosis. Similarly, detection of *B. henselae* DNA by metagenomic sequencing supports, but is not required for, bartonellosis diagnosis. Metagenomic sequencing did not provide significant diagnostic value. Metagenomic sequencing of cell-free DNA for infectious agents can also incur increased cost and turnaround time compared to serologic testing for *Bartonella* spp. In the case presented here, serology was sufficient to make the diagnosis of bartonellosis and was used to make diagnostic and treatment decisions. The metagenomic sequencing resulted several days later after patient discharge and did not impact management. Thus, the clinical utility of such testing should be carefully considered before ordering to avoid unnecessary costs and diagnostic redundancy.

Histopathologic investigation in the case presented here did not include direct observation of microorganisms but illustrates one of many patterns of glomerular injury that may be seen in association with bartonellosis. Several cases of *Bartonella*-associated ANCA-positive glomerulonephritis have been reported, highlighting the risk of misdiagnosis of vasculitis rather than infection-related glomerulonephritis (11, 13, 15). Studies have shown that while the rate of ANCA positivity can range from 18% to 33% in infective endocarditis cases, positivity can be as high as 60–75% in *Bartonella*-associated glomerulonephritis (15, 22, 23). Histological features on kidney biopsy show slightly higher occurrence of crescents within glomeruli in *Bartonella* infections compared to other microorganisms that cause infective endocarditis (83% vs 61%) (23). C3-dominant immunofluorescence staining with frequent C1q and IgM staining is common. IgA positive staining is seen most often in staphylococcal infection-associated glomerulonephritis (22, 24). Mesangial and capillary wall electron-dense deposits occur with near equal incidence in both *Bartonella*-associated glomerulonephritis and glomerulonephritis caused by other microorganisms (23). Subepithelial humps occur in a small percentage of cases in both *Bartonella*-associated and other infection-related glomerulonephritis.

Given that *Bartonella*-associated glomerulonephritis often occurs in the setting of culture-negative endocarditis, it is important to consider infective endocarditis when evidence of glomerulonephritis and *Bartonella* infection is present. The 2023 Duke-ISCVID criteria for diagnosing infective endocarditis include positive *Bartonella* serologies as a major diagnostic criterion, and immune-complex-mediated glomerulonephritis itself is a minor criterion (25). In this case, however, insufficient criteria were met to make the diagnosis of definite infective endocarditis. Features meeting criteria for diagnosis of infective endocarditis in this case were the microbiologic evidence of *B. henselae* infection, immune-complex-mediated glomerulonephritis, and fever (one major and two minor criteria). Overt signs of cardiac involvement (e.g., vegetations or other valvular abnormalities seen on cardiac imaging studies) were absent. It should be noted that this does not exclude a potential role for endocarditis in the pathophysiology of this patient’s infection. A subclinical and/or remote *B. henselae* endocarditis could have facilitated dissemination of the organism and resulted in glomerulonephritis. From a diagnostic standpoint, however, this case shows that *B. henselae* should still be considered in cases of glomerulonephritis even in the absence of a diagnosis of a concurrent endocarditis.

Glomerulonephritis associated with *Bartonella* infection is challenging to diagnose from both the clinical and histopathological perspectives. Delayed diagnosis and treatment could result in significant complications for immunosuppressed patients. As such, consideration of bartonellosis should be prioritized in cases of glomerulonephritis in patients with epidemiological links to *Bartonella*, especially in immunosuppressed individuals such as solid organ transplant recipients.

## References

[1] Mira P, Theel ES. 2024. Update on common Bartonella infections. Clin Microbiol Newsl 47:1–8. doi:10.1016/j.clinmicnews.2024.05.002

[2] Okaro U, Addisu A, Casanas B, Anderson B. 2017. Bartonella species, an emerging cause of blood-culture-negative endocarditis. Clin Microbiol Rev 30:709–746. doi:10.1128/CMR.00013-17 28490579 PMC5475225

[3] Guptill L, Wu C-C, HogenEsch H, Slater LN, Glickman N, Dunham A, Syme H, Glickman L. 2004. Prevalence, risk factors, and genetic diversity of Bartonella henselae infections in pet cats in four regions of the United States. J Clin Microbiol 42:652–659. doi:10.1128/JCM.42.2.652-659.2004 14766832 PMC344466

[4] Psarros G, Riddell J, Gandhi T, Kauffman CA, Cinti SK. 2012. Bartonella henselae infections in solid organ transplant recipients: report of 5 cases and review of the literature. Medicine (Baltimore) 91:111–121. doi:10.1097/MD.0b013e31824dc07a 22391473

[5] Bos F, Chauveau B, Ruel J, Fontant G, Campistron E, Meunier C, Jambon F, Moreau K, Delmas Y, Couzi L, Korbi S, Charrier M, Viallard J-F, Luciani L, Merville P, Lazaro E, Kaminski H. 2022. Serious and atypical presentations of Bartonella henselae infection in kidney transplant recipients. Open Forum Infect Dis 9:ofac059. doi:10.1093/ofid/ofac059 35211636 PMC8863078

[6] Andrian T, Novel-Catin E, Triffault-Fillit C, Rabeyrin M, Barba C, Koppe L, Fouque D. 2022. Crescentic glomerulonephritis with anti-PR3 ANCA associated with Bartonella henselae infective endocarditis. Clin Kidney J 15:1966–1968. doi:10.1093/ckj/sfac117 36158150 PMC9494545

[7] Babiker A, El Hag MI, Perez C. 2018. Bartonella infectious endocarditis associated with cryoglobulinemia and multifocal proliferative glomerulonephritis. Open Forum Infect Dis 5:ofy186. doi:10.1093/ofid/ofy186 30151411 PMC6101537

[8] Bookman I, Scholey JW, Jassal SV, Lajoie G, Herzenberg AM. 2004. Necrotizing glomerulonephritis caused by Bartonella henselae endocarditis. Am J Kidney Dis 43:e25–e30. doi:10.1053/j.ajkd.2003.10.027 14750122

[9] Guo S, Pottanat ND, Herrmann JL, Schamberger MS. 2022. Bartonella endocarditis and diffuse crescentic proliferative glomerulonephritis with a full-house pattern of immune complex deposition. BMC Nephrol 23:181. doi:10.1186/s12882-022-02811-w 35549887 PMC9097344

[10] Khalighi MA, Nguyen S, Wiedeman JA, Palma Diaz MF. 2014. Bartonella endocarditis-associated glomerulonephritis: a case report and review of the literature. Am J Kidney Dis 63:1060–1065. doi:10.1053/j.ajkd.2013.10.058 24332768

[11] Raybould JE, Raybould AL, Morales MK, Zaheer M, Lipkowitz MS, Timpone JG, Kumar PN. 2016. Bartonella endocarditis and Pauci-immune glomerulonephritis: a case report and review of the literature. Infect Dis Clin Pract (Baltim Md) 24:254–260. doi:10.1097/IPC.0000000000000384 27885316 PMC5098464

[12] Shaikh G, Gosmanova EO, Rigual-Soler N, Der Mesropian P. 2020. Systemic bartonellosis manifesting with endocarditis and membranoproliferative glomerulonephritis. J Investig Med High Impact Case Rep 8:2324709620970726. doi:10.1177/2324709620970726 PMC7656866 33155512

[13] Teoh LSG, Hart HH, Soh MC, Christiansen JP, Bhally H, Philips MS, Rai-Chaudhuri DS. 2010. Bartonella henselae aortic valve endocarditis mimicking systemic vasculitis. BMJ Case Rep 2010:bcr0420102945. doi:10.1136/bcr.04.2010.2945 PMC3030181 22791485

[14] Vercellone J, Cohen L, Mansuri S, Zhang PL, Kellerman PS. 2018. Bartonella endocarditis mimicking crescentic glomerulonephritis with PR3-ANCA positivity. Case Rep Nephrol 2018:9607582. doi:10.1155/2018/9607582 30210883 PMC6120290

[15] Yoshifuji A, Hibino Y, Komatsu M, Yasuda S, Hosoya K, Kobayashi E, Baba Y, Hirose S, Hashiguchi A, Kanno Y, Ryuzaki M. 2021. Glomerulonephritis caused by Bartonella spp. infective endocarditis: the difficulty and importance of differentiation from anti-neutrophil cytoplasmic antibody-related rapidly progressive glomerulonephritis. Intern Med 60:1899–1906. doi:10.2169/internalmedicine.5608-20 33456034 PMC8263179

[16] Singhania G, Singhania N. 2019. Do we care if you have a cat? Bartonella infection related glomerulonephritis with no endocarditis. Infez Med 27:441–444. 31846996

[17] Chaudhry AR, Chaudhry MR, Papadimitriou JC, Drachenberg CB. 2015. Bartonella henselae infection‐associated vasculitis and crescentic glomerulonephritis leading to renal allograft loss. Transpl Infect Dis 17:411–417. doi:10.1111/tid.12376 25753276

[18] Pischel L, Radcliffe C, Vilchez GA, Charifa A, Zhang X-C, Grant M. 2021. Bartonellosis in transplant recipients: a retrospective single center experience. World J Transplant 11:244–253. doi:10.5500/wjt.v11.i6.244 34164299 PMC8218350

[19] Agan BK, Dolan MJ. 2002. Laboratory diagnosis of Bartonella infections. Clin Lab Med 22:937–962. doi:10.1016/s0272-2712(02)00017-3 12489289

[20] Liesman RM, Pritt BS, Maleszewski JJ, Patel R. 2017. Laboratory diagnosis of infective endocarditis. J Clin Microbiol 55:2599–2608. doi:10.1128/JCM.00635-17 28659319 PMC5648697

[21] Rodino KG, Stone E, Saleh OA, Theel ES. 2019. The brief case: Bartonella henselae endocarditis—a case of delayed diagnosis. J Clin Microbiol 57:00114–00119. doi:10.1128/JCM.00114-19 PMC6711904 31451567

[22] Mahr A, Batteux F, Tubiana S, Goulvestre C, Wolff M, Papo T, Vrtovsnik F, Klein I, Iung B, Duval X, the IMAGE Study Group. 2014. Brief report: prevalence of antineutrophil cytoplasmic antibodies in infective endocarditis. Arthritis Rheumatol Hoboken NJ 66:1672–1677. doi:10.1002/art.38389 24497495

[23] Kitamura M, Dasgupta A, Henricks J, Parikh SV, Nadasdy T, Clark E, Bazan JA, Satoskar AA. 2023. Clinicopathological differences between Bartonella and other bacterial endocarditis-related glomerulonephritis - our experience and a pooled analysis. Front Nephrol 3:1322741. doi:10.3389/fneph.2023.1322741 38288381 PMC10823370

[24] Nasr SH, Radhakrishnan J, D’Agati VD. 2013. Bacterial infection–related glomerulonephritis in adults. Kidney Int 83:792–803. doi:10.1038/ki.2012.407 23302723

[25] Fowler VG Jr, Durack DT, Selton-Suty C, Athan E, Bayer AS, Chamis AL, Dahl A, DiBernardo L, Durante-Mangoni E, Duval X, Fortes CQ, Fosbøl E, Hannan MM, Hasse B, Hoen B, Karchmer AW, Mestres CA, Petti CA, Pizzi MN, Preston SD, Roque A, Vandenesch F, van der Meer JTM, van der Vaart TW, Miro JM. 2023. The 2023 Duke-International Society for cardiovascular infectious diseases criteria for infective endocarditis: updating the modified duke criteria. Clin Infect Dis 77:518–526. doi:10.1093/cid/ciad271 37138445 PMC10681650

